# Cannabidiol prevents cognitive and social deficits in a male rat model of Alzheimer’s disease through CB1 activation and inflammation modulation

**DOI:** 10.1038/s41386-025-02213-0

**Published:** 2025-08-26

**Authors:** Roni Shira Toledano, Irit Akirav

**Affiliations:** 1 School of Psychological Sciences, Department of Psychology, University of Haifa, Haifa, 3498838 Israel; 2 The Integrated Brain and Behavior Research Center (IBBRC), University of Haifa, Haifa, 3498838 Israel

**Keywords:** Cognitive ageing, Neuroscience

## Abstract

Cognitive decline is a hallmark of Alzheimer’s disease (AD). Cannabidiol (CBD), a non-intoxicating phytocannabinoid with immunomodulatory properties, shows promise in alleviating AD symptoms. This study examined the effects of chronic CBD treatment in a male rat model of sporadic AD induced by intracerebroventricular streptozotocin (ICV-STZ) and explored its impact on neuroinflammatory genes and cannabinoid signaling. STZ rats showed impaired performance in object location and recognition tasks, along with reduced social behavior. STZ exposure also affected AD-related hippocampal markers, leading to increased levels of amyloid β-protein (Aβ) and tau phosphorylation (p-Tau) and elevated mRNA levels of triggering receptor expressed on myeloid cells 2 (TREM2) and apolipoprotein E4 (APOEε4). Additionally, STZ increased hippocampal neuroinflammatory markers, including mRNA levels of Tumor Necrosis Factor α (TNF-α), nuclear factor kappa B subunit 1 (NF-κB1), and interleukin (IL)-1β. It also altered cannabinoid receptor expression, with cannabinoid receptor 1 (cnr1) and 2 (cnr2) genes upregulated in the dentate gyrus (DG), whereas in the CA1, cnr2 was upregulated and cnr1 downregulated. Chronic CBD treatment restored the STZ-induced behavioral deficits, reduced neuroinflammatory marker expression, and mitigated AD-associated changes. Importantly, the CB1 receptor antagonist AM251, but not CB2 antagonist AM630, blocked the beneficial effects of CBD on performance in object location and social tasks in STZ-treated rats, highlighting CB1 receptor activation as a key mechanism. These findings suggest that CBD holds promise as a therapeutic agent for inflammation-induced AD, with the potential to ameliorate cognitive deficits and prevent disease onset through mechanisms involving CB1 receptor activation and modulation of neuroinflammation.

## Introduction

Alzheimer’s disease (AD) is marked by progressive cognitive decline, amyloid β-protein (Aβ) accumulation, tau pathology, neuronal death, and brain inflammation, leading to neuropsychiatric symptoms like depression, anxiety, and social withdrawal [1, 2].

Neuroinflammation, marked by elevated interleukins (IL-1, IL-6), Tumor Necrosis Factor α (TNF-α), and nuclear factor kappa B subunit 1 (NF-kB1), is a central feature of AD; it is frequently found in postmortem AD brains [3–5], often preceding Aβ and tau pathology [6]. Genetic factors like apolipoprotein E4 (APOEε4) and triggering receptor expressed on myeloid cells 2 (TREM2) also regulate inflammatory responses [7, 8].

The intracerebroventricular (ICV) injection of streptozotocin (STZ) in rats effectively mimics key features of sporadic AD, inducing persistent neuroinflammation, glial activation, reduced hippocampal neurogenesis, and spatial memory deficits [9–11]. In transgenic AD models, ICV-STZ exacerbates neuroinflammation, cognitive deficits, plaque pathology, and tau hyperphosphorylation, increasing AD vulnerability [12–14]. The ICV-STZ model is induced in mature rats, rather than aged ones, to isolate Alzheimer’s-like symptoms from changes associated with aging [11].

Cannabidiol (CBD) shows promise in alleviating AD symptoms by immunomodulatory and neuroprotective properties in neurodegenerative diseases [15–18]. CBD prevents learning and memory deficits in Aβ-injected mice [19] and improves spatial learning and anxiety in female APPxPS1 mice [20].

CBD prevents neuroinflammation through multiple mechanisms, including the reduction of pro-inflammatory markers such as TNF-α, NF-κB1, IL-1β, and inducible nitric oxide synthase [21, 22]. This effect is partly mediated by inhibiting fatty acid amide hydrolase (FAAH) and modulation of cannabinoid receptors. Specifically, CBD acts as a CB2 partial agonist and a CB1 negative allosteric modulator, influencing receptor function without direct activation [23, 24].

This study investigated CBD’s potential to prevent cognitive impairments and AD-related markers in the ICV-STZ model. As this is an initial study aimed at investigating the underlying mechanisms of CBD’s preventive effects, only male subjects were used to minimize variability and ensure consistency. Also, Sprague-Dawley (SD) female rats display differences in some AD typical biomarkers compared to those observed in humans [25].

Additionally, we examined CBD’s effects on hippocampal neuroinflammatory markers and cannabinoid receptor expression, and the role of CB1r and CB2r antagonists (AM251 and AM630) in mediating CBD’s therapeutic outcomes. With an established safety profile, limited side effects, and accessibility, CBD has the potential for rapid clinical translation as a candidate treatment for AD.

## Materials and methods

For elaborated procedures see supplementary information.

### Animals

Adult male SD rats (Envigo Laboratories, Jerusalem) were used. The study was approved by the University of Haifa Ethics and Animal Care Committee (UoH-IL-2207-164-4) and adhered to NIH guidelines for minimizing pain and discomfort.

### Pharmacology

Rats were chronically injected with vehicle, CBD (0.1 mg/kg or 1 mg/kg), the CB1r antagonist AM251 (0.3 mg/kg), or the CB2r antagonist AM630 (1 mg/kg) (Cayman Chemical, USA) daily for two weeks. All drugs were freshly prepared and administered intraperitoneally (i.p.) at a volume of 1 ml/kg. CBD was dissolved in vehicle containing 2% Tween-80 and 98% saline. AM251 and AM630 were prepared in vehicle containing 5% dimethylsulfoxide (DMSO), 5% Tween-80, and 90% saline. Doses were based on previous publications [26–29].

### Surgical procedure and microinjection

The surgical procedure followed the protocol described in reference (25). Rats were anesthetized with ketamine/domitor (75/0.5 mg/kg, subcutaneously) and placed in a stereotaxic apparatus. On day 0, an ICV injection of STZ (3 mg/kg; 10 μL volume; 2 μL/min) (Sigma-Aldrich, Rehovot, Israel) or aCSF (dissolved in 10 μL DDW) (hello bio, Bristol, UK) was administered into the left ventricle, using a Hamilton syringe (Hamilton Co., USA) (coordinates: AP − 0.8 mm, ML + 1.5 mm, DV − 3.6 mm). The syringe was held for 10 min post-injection to prevent reflux. STZ dosing was based on [12, 30].

### Behavioral tests

The open field (OF) test assessed general locomotor function (total distance, cm) and novelty-induced anxiogenic behavior (time in arena center, first 5 min). The Object Location (OL) and Novel Object Recognition (NOR) tests, with an inter-trial interval (ITI) of 5 min, were used to measure spatial and visual recognition, as well as working memory. We assessed total exploration time (s) and the mean discrimination index (DI), calculated as TN/TN + TF (TN = novel place/object exploration time, TF = familiar place/object exploration time) in the test phase. The Social Interaction Test (SIT) assessed social and non-social behaviors. Time (s) spent on each behavior was measured, and the sociability index was calculated as the time spent engaging in social behaviors divided by the total test time (5 min).

### Western blotting (WB)

Protein expression levels of Aβ, Tau, and p-Tau were assessed by Western blot, as previously described [31]. Protein quantification was performed using a BCA assay, and β-actin served as a loading control.

### Quantitative real-time PCR (qRT-PCR)

cDNA preparation and qRT-PCR were performed using standard methodology as previously described [32, 33]. We examined mRNA gene expression levels of TNF-α, NF-κB1, IL-1β, IL-6, CB1r, CB2r, ApoEɛ4, and TREM2 (Table S1).

#### Immunohistochemistry procedure

Anesthetized rats were perfused, and brains were fixated and frozen at −80 °C until sectioned with a cooled cryostat (40 μm) (Leica, CM1900). Sections were washed, blocked, and incubated overnight with primary antibodies against p-Tau (pS396) and Aβ. Following PBS washes, Alexa Fluor-conjugated secondary antibodies were applied. Sections were mounted with antifade medium and stored at 4 °C.

### Statistical analysis

Results are presented as means ± SEM. Data were analyzed using one-way/two-way ANOVA and Pearson correlation, with post hoc Tukey’s test where applicable. Normality was assessed via Kolmogorov–Smirnov and Shapiro–Wilk tests. Statistical significance was set at *p* ≤ 0.05, and analysis was performed in SPSS 27 (IBM).

For experimental design see Fig. S1.

## Results

### Effects of chronic CBD administration on behavior of rats with ICV-STZ-Induced AD

**Fig. 1:**
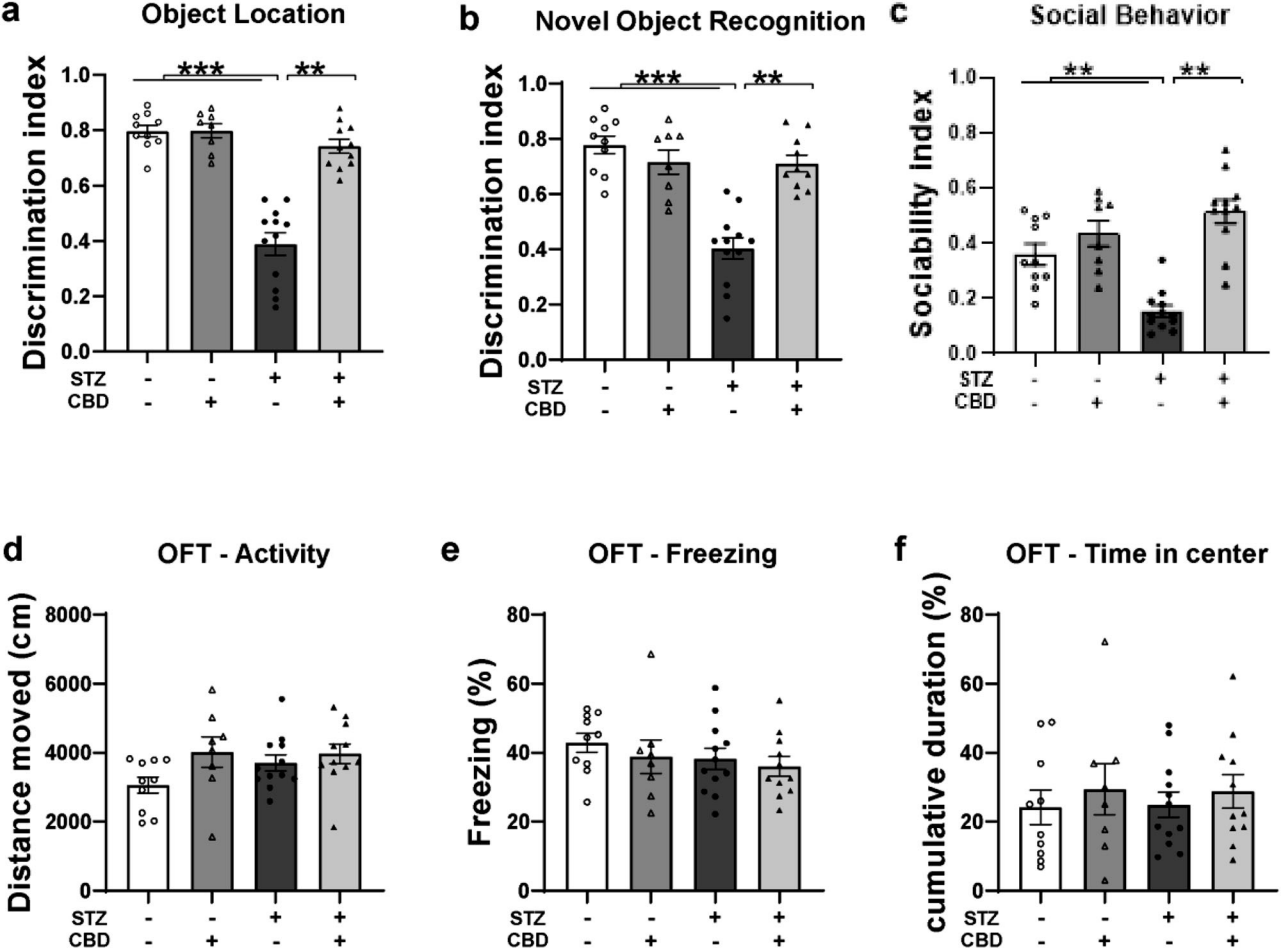
Effects of Chronic CBD Administration on the Behavior of Rats with ICV-STZ-Induced AD. Compared to all other experimental groups, animals in the STZ-Veh group showed impaired performance in the **a** object location task, **b** the novel object recognition task, and **c** decreased sociability in the social interaction task. No differences were observed between the groups in **d** distance traveled, **e** freezing levels, or **f** time spent in the center during the open field test. (*n* = 8–10 per group), **p < 0.01, ***p < 0.001.

In the OL task (Fig. 1a), a two-way ANOVA revealed significant effects of STZ [F(1,40) = 55.512, *p* < 0.001], CBD [F(1,40) = 32.249, *p* < 0.001], and the STZ×CBD interaction [F(1,40) = 31.977, *p* < 0.001]. Post-hoc comparisons indicated a lower DI in the STZ+Veh group compared to the STZ + CBD (*p* < 0.01), aCSF+Veh (*p* < 0.001), and aCSF+CBD (*p* < 0.001) groups.

In the NOR task (Fig. 1b), a two-way ANOVA revealed significant effects of STZ [F(1,40) = 26.373, *p* < 0.001], CBD [F(1,40) = 11.05, *p* < 0.01], and the STZ×CBD interaction [F(1,40) = 24.936, *p* < 0.001]. Post-hoc comparisons showed a lower DI in the STZ+Veh group compared to the STZ + CBD (*p* < 0.01), aCSF+Veh (*p* < 0.001), and aCSF+CBD (*p* < 0.001) groups.

We also measured the total exploration time in the cognitive tests (Fig. S2).

In the SIT (Fig. 1c), a two-way ANOVA revealed significant effects of STZ [F(1,40) = 19.697, *p* < 0.01], CBD [F(1,40) = 34.394, *p* < 0.001], and the STZ×CBD interaction [F(1,40) = 14.799, *p* < 0.001]. Post-hoc comparisons showed a lower sociability index in the STZ+Veh group compared to the STZ + CBD (*p* < 0.01), aCSF+Veh (*p* < 0.01), and aCSF+CBD (*p* < 0.01) groups.

These findings suggest that CBD prevented STZ-induced impairment in the OL and NOR tasks, as well as in social behavior. Individual social behaviors are presented in Fig. S3.

In the OFT, no significant effects were observed for distance traveled (Fig. 1d), freezing levels (Fig. 1e), and time spent in the center (Fig. 1f) (see Supplementary Materials for full statistical details).

In a preliminary study, we examined the effect of two low doses of CBD (0.1 mg/kg, 1 mg/kg) on performance in emotional and cognitive tests in STZ-treated animals. We found similar therapeutic effects for both doses (Fig. S4); therefore, we proceeded with the lower dose of 0.1 mg/kg.

### Effects of chronic CBD administration on hippocampal AD-related markers in rats with ICV-STZ-Induced AD

We assessed protein levels in the CA1 and DG regions.

#### Aβ protein and p-Tau

**Fig. 2:**
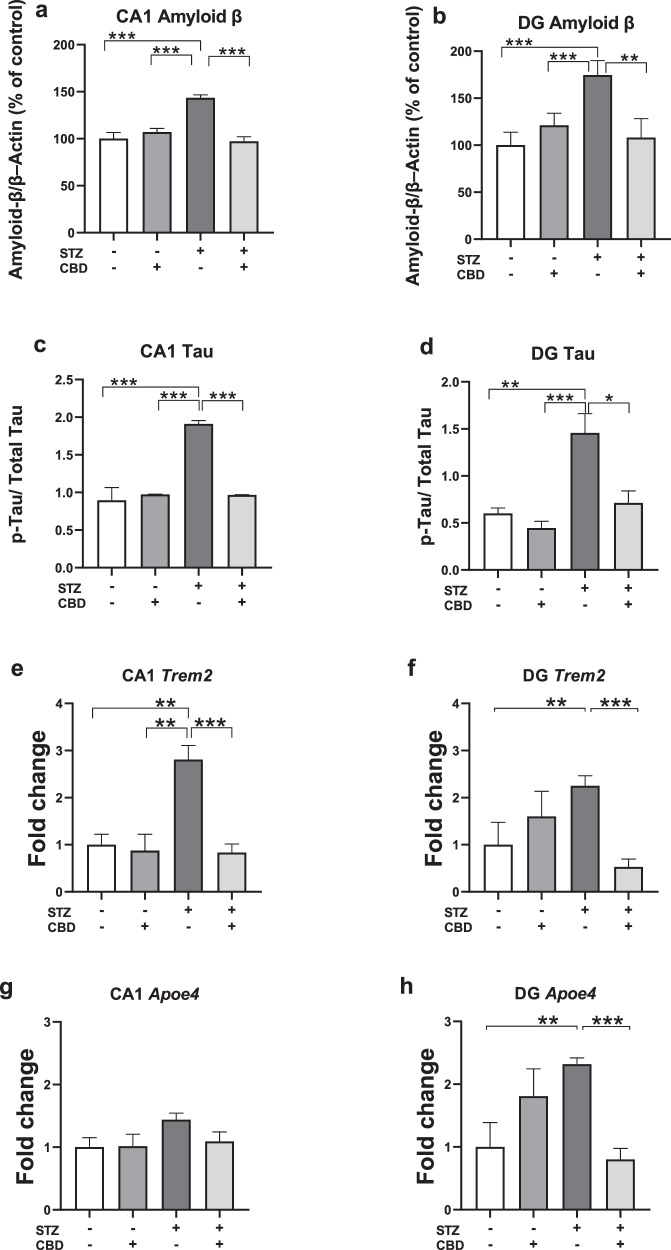
Effects of Chronic CBD Administration on AD-Related Markers in the CA1 and DG of Rats with ICV-STZ-Induced AD. Compared to all other experimental groups, animals in the STZ-Veh group exhibited increased levels of Amyloid β protein in the CA1 **a** and DG **b**, as well as increased p-Tau in the CA1 **c** and DG **d**. Compared to all other experimental groups, animals in the STZ-Veh group showed increased levels of TREM2 mRNA in the CA1 **e**; animals in the STZ-Veh group exhibited higher mRNA levels of TREM2 in the DG compared to the aCSF-Veh and the STZ-CBD groups **f**. There were no differences between the groups in ApoEɛ4 mRNA levels in the CA1 **g**; animals in the STZ-Veh group exhibited higher mRNA levels of ApoEɛ4 in the DG compared to the aCSF-Veh and the STZ-CBD groups **h**. (*n* = 5-8 per group), *p < 0.05, **p < 0.01, ***p < 0.001.

In the CA1, a two-way ANOVA revealed significant effects on Aβ protein levels (Fig. 2a) and p-Tau (Fig. 2c) of STZ [Aβ: F(1,24) = 10.44, *p* < 0.05; Tau: F(1,24) = 288.79, *p* < 0.001], CBD [Aβ: F(1,24) = 14.036, *p* < 0.001; Tau: F(1,24) = 214.423, *p* < 0.001], and the STZ×CBD interaction [Aβ: F(1,24) = 26.16, *p* < 0.001; Tau: F(1,24) = 295.315, *p* < 0.001]. Post-hoc comparisons showed a significant increase in Aβ protein and p-Tau in the STZ+Veh group compared to the STZ + CBD (Aβ: *p* < 0.001; Tau: *p* < 0.001), aCSF+Veh (Aβ: *p* < 0.001; Tau: *p* < 0.001), and aCSF+CBD (Aβ: *p* < 0.001; Tau: *p* < 0.001) groups.

In the DG, a two-way ANOVA revealed significant effects on Aβ protein levels (Fig. 2b) and p-Tau (Fig. 2d) of STZ [Aβ: F(1,28) = 5.276, *p* < 0.05; Tau: F(1,21) = 34.059, *p* < 0.001], CBD [Aβ: F(1,28) = 6.137, *p* < 0.05; Tau: F(1,21) = 19.544, *p* < 0.001], and the STZ×CBD interaction [Aβ: F(1,28) = 9.861, *p* < 0.01; Tau: F(1,21) = 6.087, *p* < 0.05]. Post-hoc comparisons showed a significant increase in Aβ protein and p-Tau in the STZ+Veh group compared to the STZ + CBD (Aβ: *p* < 0.01; Tau: *p* < 0.05), aCSF+Veh (Aβ: *p* < 0.001; Tau: *p* < 0.01), and aCSF+CBD (Aβ: *p* < 0.05; Tau: *p* < 0.001) groups.

These findings suggest that CBD prevented the STZ-induced increase in CA1- and DG-Aβ and CA1- and DG-p-Tau protein levels.

Representative Western blot images are provided in Supplementary Fig. S5.

#### TREM2

In the CA1, a two-way ANOVA revealed significant effects of STZ [F(1,21) = 7.125, *p* < 0.05], CBD [F(1,21) = 13.234, *p* < 0.01], and the STZ×CBD interaction [F(1,21) = 8.497, *p* < 0.01] (Fig. 2e). Post-hoc comparisons indicated a significant increase in TREM2 mRNA in the STZ+Veh group compared to the STZ + CBD (*p* < 0.001), aCSF+Veh (*p* < 0.01) and aCSF+CBD (*p* < 0.01) groups.

In the DG, a two-way ANOVA revealed a significant effect of STZ×CBD interaction [F(1,20) = 25.503, *p* < 0.001] (Fig. 2f) with no significant effects of STZ [F(1,20) = 0.079, ns] or CBD [F(1,20) = 2.283, ns]. Post-hoc comparisons showed a significant increase in TREM2 mRNA in the STZ+Veh group compared to the aCSF+Veh (*p* < 0.01) and STZ + CBD (*p* < 0.001) groups.

#### ApoEɛ4

In the CA1, a two-way ANOVA revealed no significant effects of STZ [F(1,21) = 0.1.958, ns], CBD [F(1,21) = 0.701, ns], or the STZ×CBD interaction [F(1,21) = 0.88, ns] (Fig. 2g).

In the DG, two-way ANOVA revealed significant effects of STZ×CBD interaction [F(1,20) = 18.13, *p* < 0.001] with no significant effect of STZ [F(1,20) = 0.106, ns] or CBD [F(1,20) = 1.612, ns] (Fig. 2h). Post-hoc comparisons showed a significant increase in ApoEɛ4 mRNA in the STZ+Veh group compared to the aCSF+Veh (*p* < 0.01) and STZ-CBD (*p* < 0.001) groups.

These findings suggest that CBD prevented the STZ-induced increase in CA1- and DG-TREM2 and DG- ApoEɛ4 mRNA levels.

Pearson bivariate correlation tests were conducted to examine the associations between AD-like behaviors and measures of mRNA and protein levels in the CA1 and DG (Table S2).

### Effects of chronic CBD administration on mRNA expression of neuroinflammatory markers and CB1/CB2 receptors in the CA1 and DG of rats with ICV-STZ-Induced AD

#### TNF-α

**Fig. 3:**
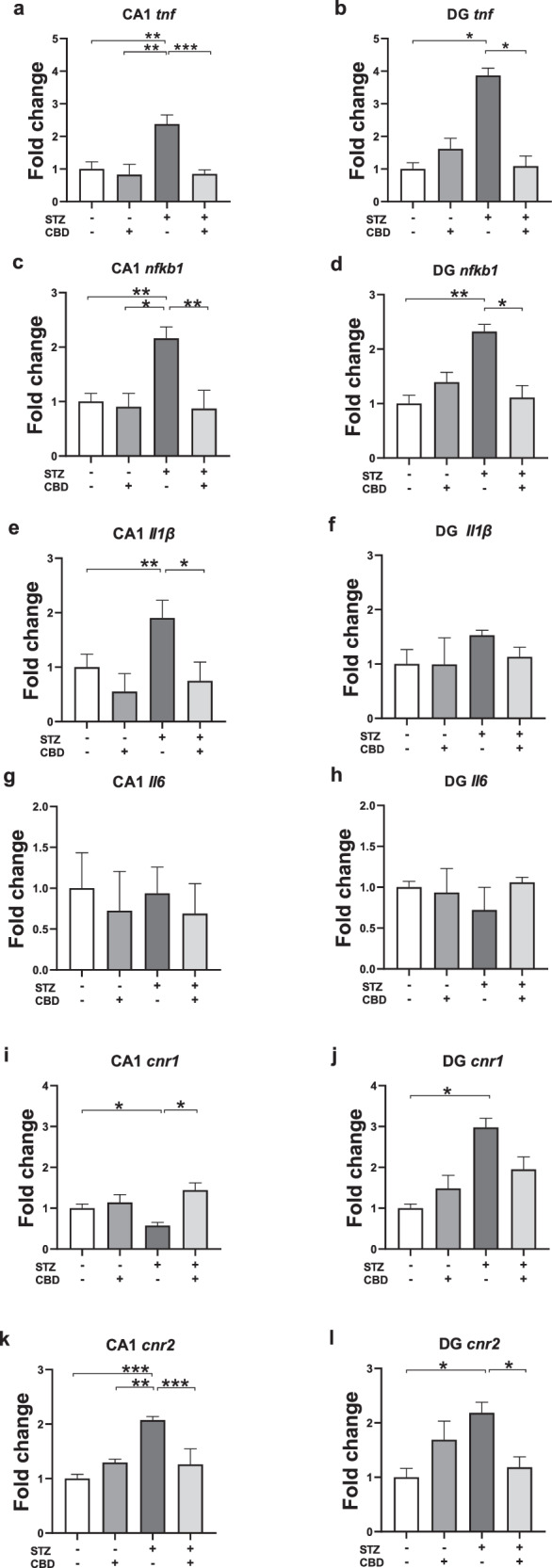
Effects of Chronic CBD Administration on mRNA Expression of Neuroinflammatory Markers and CB1/CB2 Receptors in the CA1 and DG of Rats with ICV-STZ-Induced AD. Animals in the STZ-Veh group exhibited higher mRNA levels of TNFα in the CA1 compared to all other experimental groups **a** and compared to the aCSF-Veh and STZ-CBD groups in the DG **b**. Animals in the STZ-Veh group exhibited higher mRNA levels of NF-kB1 in the CA1 compared to all other experimental groups **c** and compared to the aCSF-Veh and STZ-CBD groups in the DG **d**. Animals in the STZ-Veh group showed elevated IL-1β mRNA levels compared to the aCSF-Veh and STZ-CBD groups in the CA1 **e**; no differences between the groups were observed in IL-1β mRNA levels in the DG **f**. No differences between the groups were observed in IL-6 mRNA levels in the CA1 **g** nor in the DG **h**. Compared to the aCSF-Veh and STZ-CBD groups, animals in the STZ-Veh group exhibited reduced CB1r mRNA levels in the CA1 **i** and increased CB1r mRNA levels in the DG **j**. **k**, **l** Animals in the STZ-Veh group exhibited higher mRNA levels of CB2r mRNA levels in the CA1 compared to all other experimental groups **k** and compared to the aCSF-Veh and the STZ-CBD groups in the DG. (*n* = 5–8 per group), *p < 0.05, **p < 0.01, ***p < 0.001.

In the CA1, a two-way ANOVA revealed significant effects of STZ [F(1,27) = 7.415, *p* < 0.01], CBD [F(1,27) = 13.908, *p* < 0.001], and the STZ×CBD interaction [F(1,27) = 6.585, *p* < 0.05] (Fig. 3a). Post-hoc comparisons showed a significant increase in TNF-α mRNA in the STZ+Veh group compared to the STZ + CBD (*p* < 0.001), aCSF+Veh (*p* < 0.01), and aCSF+CBD (*p* < 0.001) groups.

In the DG, a two-way ANOVA revealed significant effects of STZ×CBD interaction [F(1,20) = 12.278, *p* < 0.01] (Fig. 3b) with no significant effect of STZ [F(1,20) = 3.677, ns] or CBD [F(1,20) = 2.486, ns]. Post-hoc comparisons showed a significant increase in TNF-α mRNA in the STZ+Veh group compared to the STZ + CBD (*p* < 0.05) and aCSF+Veh (*p* < 0.05) groups.

These findings suggest that CBD prevented the STZ-induced increase of CA1- and DG-TNF-α.

#### NF-κB1

In the CA1, a two-way ANOVA revealed significant effects of STZ [F(1,24) = 4.649, *p* < 0.05], CBD [F(1,24) = 8.858, *p* < 0.01], and the STZ×CBD interaction [F(1,24) = 5.664, *p* < 0.05] (Fig. 3c). Post-hoc comparisons showed a significant increase in NF-κB1 mRNA in the STZ+Veh group compared to the STZ + CBD (*p* < 0.01), aCSF+Veh (*p* < 0.01), and aCSF+CBD (*p* < 0.05) groups.

In the DG, a two-way ANOVA revealed a significant effect of STZ×CBD interaction [F(1,20) = 11.668, *p* < 0.01] (Fig. 3d) with no significant effects of STZ [F(1,20) = 3.901, ns] or CBD [F(1,20) = 1.672, ns]. Post-hoc comparisons showed a significant increase in NF-κB1 mRNA in the STZ+Veh group compared to the STZ + CBD (*p* < 0.05) and aCSF+Veh (*p* < 0.05) groups.

These findings suggest that CBD prevented the STZ-induced increase in CA1- and DG-NF-κB1.

#### IL-1β

In the CA1, a two-way ANOVA revealed significant effects of STZ [F(1,23) = 4.784, *p* < 0.05], and CBD [F(1,23) = 12.449, *p* < 0.01], with no significant STZ×CBD interaction [F(1,23) = 0.622, ns] (Fig. 3e). Post-hoc comparisons showed a significant increase in IL-1β mRNA levels in the STZ+Veh group compared to both the STZ + CBD (*p* < 0.05) and aCSF+Veh (*p* < 0.01) groups, suggesting that CBD prevented the STZ-induced increase in CA1- IL-1β.

In the DG, a two-way ANOVA revealed no significant effects of STZ [F(1,20) = 0.64, ns], CBD [F(1,20) = 0.196, ns], or the STZ×CBD interaction [F(1,20) = 0.183, ns] (Fig. 3f).

#### IL-6

In the CA1 (Fig. 3g) and DG (Fig. 3h), a two-way ANOVA revealed no significant effects of STZ [CA1: F(1,21) = 0.000, ns; DG: F(1,20) = 0.034, ns], CBD [CA1: F(1,21) = 0.883, ns; DG: F(1,20) = 0.084, ns], or the STZ×CBD interaction [CA1: F(1,21) = 0.044, ns; DG: F(1,20) = 0.173, ns].

#### CB1r

In the CA1, a two-way ANOVA revealed significant effects of CBD [F(1,24) = 8.535, *p* < 0.01] and the STZ×CBD interaction [F(1,24) = 4.441, *p* < 0.05], with no significant effect of STZ [F(1,24) = 1.633, ns] (Fig. 3i). Post-hoc comparisons showed a significant decrease in CB1r mRNA in the STZ+Veh group compared to the STZ + CBD (*p* < 0.01) and aCSF+Veh (*p* < 0.05) groups, suggesting that CBD prevented the STZ-induced decrease in CA1-CB1r.

In the DG, a two-way ANOVA revealed a significant effect of STZ [F(1,20) = 8.199, *p* < 0.05], with no significant effects of CBD [F(1,20) = 0.003, ns], or STZ×CBD interaction [F(1,20) = 2.939, ns] (Fig. 3j). Post-hoc comparisons indicated a significant increase in CB1r mRNA in the STZ+Veh group compared to the aCSF+Veh group (*p* < 0.05), suggesting an effect of the STZ model.

#### CB2r

In the CA1, a two-way ANOVA revealed significant effects of STZ [F(1,26) = 7.798, *p* < 0.05], CBD [F(1,26) = 5.909, *p* < 0.05], and the STZ×CBD interaction [F(1,26) = 18.454, *p* < 0.001] (Fig. 5f). Post-hoc comparisons indicated a significant increase in CB2r mRNA in the STZ+Veh group compared to the STZ + CBD (*p* < 0.001), aCSF+Veh (*p* < 0.001), and aCSF+CBD (*p* < 0.01) groups.

In the DG, a two-way ANOVA revealed a significant effect of STZ×CBD interaction [F(1,20) = 10.758, *p* < 0.005] (Fig. 3l), with no significant effects of STZ [F(1,20) = 1.514, ns] or CBD [F(1,20) = 0.063, ns]. Post-hoc comparisons indicated a significant increase in CB2r mRNA in the STZ+Veh group compared to the aCSF+Veh (*p* < 0.05) and STZ + CBD (*p* < 0.05) groups.

These findings suggest that CBD prevented the STZ-induced increase in CA1- and DG-CB2r.

Pearson bivariate correlation tests were conducted to explore the associations between AD-like behaviors and mRNA expression levels of neuroinflammatory markers and CB1/CB2 genes in the CA1 and DG (Tables S3, 4).

### Effects of CB1 and CB2 receptor blockade on behavior following chronic CBD administration in rats with ICV-STZ-induced AD

Given the changes in CB1r and CB2r gene expression, we next examined the impact of receptor blockers on CBD’s therapeutic effects on behavior. The experiment followed the same procedure as the first, with the addition of AM251 (CB1r antagonist) and AM630 (CB2r antagonist), administered with or without CBD. Animals were then tested in the same behavioral assays as previously described (Fig. S1).

**Fig. 4:**
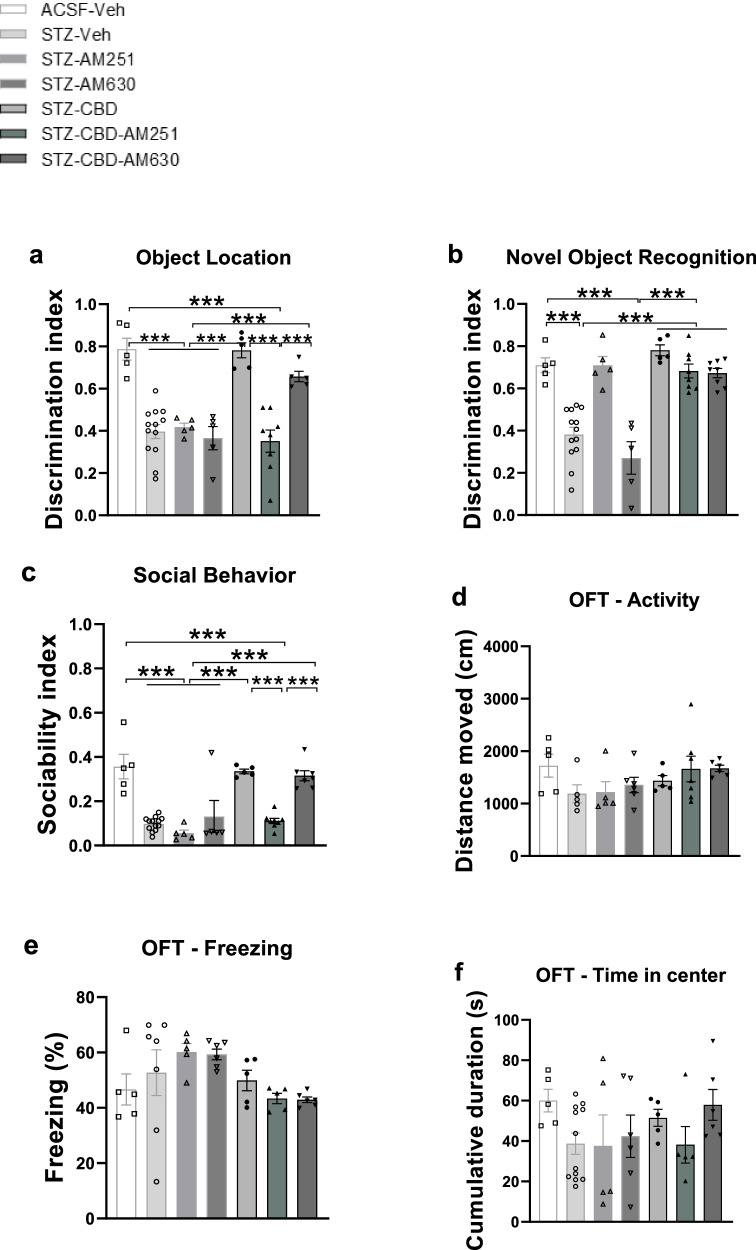
Effects of CB1 and CB2 Receptor Blockade on Behavior Following Chronic CBD Administration in Rats with ICV-STZ-Induced AD. **a** Compared to the STZ + CBD, STZ + CBD + AM630 and aCSF+Veh groups, the STZ + CBD + AM251 group showed impaired performance in the object location task. In contrast, the STZ + CBD + AM630 showed intact performance relative to the STZ+Veh, STZ + AM251, STZ + AM630, and STZ + CBD + AM251 groups. **b** Animals in the STZ + CBD + AM251 and STZ + CBD + AM630 groups showed intact performance in the novel object recognition task compared to the STZ+Veh and STZ + AM630 groups. **c** Compared to the STZ + CBD, STZ + CBD + AM630 and aCSF+Veh groups, the STZ + CBD + AM251 group showed reduced sociability in the SIT. In contrast, the STZ + CBD + AM630 group exhibited improved performance compared to the STZ+Veh, STZ + AM251, STZ + AM630, and STZ + CBD + AM251 groups. No significant differences were observed between the groups in **d** distance traveled, **e** freezing levels, or **f** time spent in the center during the open field test. (*n* = 5–10 per group), **p < 0.01, ***p < 0.001.

In the OL task (Fig. 4a), a one-way ANOVA revealed significant group effects [F(6,42) = 18.49, *p* < 0.001]. Post-hoc comparisons indicated that the DI was significantly lower in the STZ + CBD + AM251 group compared to the STZ + CBD (*p* < 0.001), STZ + CBD + AM630 (*p* < 0.001), and aCSF+Veh (*p* < 0.001) groups. Additionally, the DI in the STZ + CBD + AM630 group was significantly higher than in the STZ+Veh (*p* < 0.001), STZ + AM251 (*p* < 0.001), STZ + AM630 (*p* < 0.001), and STZ + CBD + AM251 (*p* < 0.001) groups. These findings suggest that AM251, but not AM630, blocked the effects of CBD on performance in the OL task in STZ rats.

In the NOR task (Fig. 4b), a one-way ANOVA revealed significant group effects [F(6,42) = 22.584, *p* < 0.001]. Post-hoc comparisons showed that the DI was significantly higher in the STZ + CBD + AM251 and STZ + CBD + AM630 groups compared to the STZ+Veh (*p* < 0.001) and STZ + AM630 (*p* < 0.001) groups.

We also measured total exploration time during the cognitive tests (Fig. S6).

In the SIT (Fig. 4c), a one-way ANOVA revealed significant group effects [F(6,42) = 42.968, *p* < 0.001]. Post-hoc comparisons showed that the sociability index was significantly lower in the STZ + CBD + AM251 group compared to the STZ + CBD (*p* < 0.001), STZ + CBD + AM630 (*p* < 0.001), and aCSF+Veh (*p* < 0.001) groups. Additionally, the sociability index in the STZ + CBD + AM630 group was significantly higher than in the STZ+Veh (*p* < 0.001), STZ + AM251 (p < 0.001), STZ + AM630 (*p* < 0.001), and STZ + CBD + AM251 (*p* < 0.001) groups. Individual social behaviors are presented in Fig. S7.

These findings suggest that in STZ rats, AM251—but not AM630—blocked the effects of CBD in the OL and SIT tasks, while neither antagonist blocked CBD’s effects in the NOR task. Moreover, AM251 alone appeared to enhance NOR performance in STZ rats.

In the OFT, no significant group effects were observed for distance traveled (Fig. 4d), freezing levels (Fig. 4e), or time spent in the center (Fig. 4f) (see Supplementary Materials for full statistical details).

### Effects of chronic CBD administration on Aβ and p-Tau accumulation in the hippocampus of rats with ICV-STZ-Induced AD

Immunohistochemical analysis was used to quantify Aβ-positive and p-Tau cells in the CA1 and DG.

**Fig. 5:**
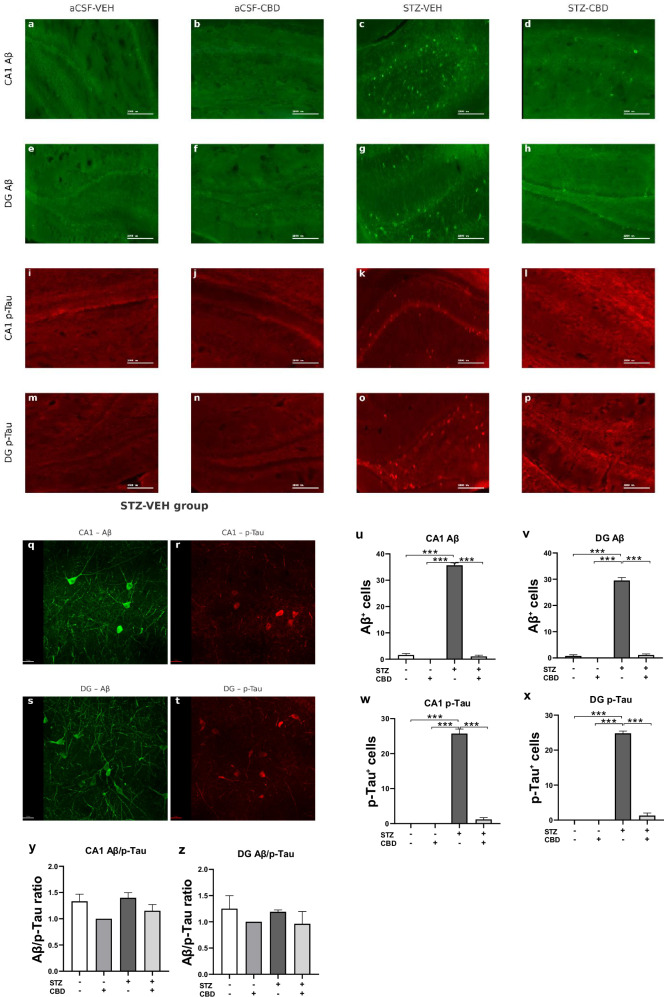
Immunohistochemical Analysis of Aβ and p-Tau in the CA1 and DG of Rats with ICV-STZ-Induced AD. **a–p** Representative images illustrating Aβ (green; **a–h**) and p-Tau (red; **i–p**) labeling in the CA1 and DG in the different experimental groups (Scale bar = 1000 μm). **q–t** Enlarged (40×) representative images of Aβ and p-Tau labeling in the STZ-VEH group: CA1 **q, r** and DG **s, t**, presented with a 30 μm scale. Panels u-z present quantification of Aβ⁺ **u, v** and p-Tau⁺ **w, x** cells in CA1 and DG. STZ administration significantly increased the number of Aβ⁺ and p-Tau⁺ cells in both regions compared to control groups. CBD co-treatment reduced the number of labeled cells in STZ-treated animals. Analysis of the Aβ/p-Tau cell ratio in CA1 **y** and DG **z** revealed no significant differences among the groups. Data are presented as mean ± SEM. Statistical analysis was performed using two-way ANOVA followed by post hoc comparisons. (*n* = 4 per group), ***p < 0.001.

A two-way ANOVA conducted on Aβ expression in the CA1 and DG revealed significant effects of STZ [CA1: F(1,16) = 837.537, *p* < 0.001; DG: F(1,16) = 520.745, *p* < 0.001], CBD [CA1: F(1,16) = 890.022, *p* < 0.001; DG: F(1,16) = 496.341, *p* < 0.001], and the STZ×CBD interaction [CA1:F(1,16) = 741.087, *p* < 0.001; DG: F(1,16) = 446.594, *p* < 0.001] (Fig. 5u–v). Consistent with the findings for Aβ, the analysis of p-Tau yielded similar results; a two-way ANOVA conducted on p-Tau expression in the CA1 and DG regions revealed significant effects of STZ [CA1: F(1,16) = 366.313, *p* < 0.001; DG: F(1,16) = 696.853, *p* < 0.001], CBD [CA1: F(1,16) = 304.539, *p* < 0.001; DG: F(1,16) = 565.753, *p* < 0.001], and the STZ×CBD interaction [CA1: F(1,16) = 304.539, *p* < 0.001; DG: F(1,16) = 565.753, *p* < 0.001] (Fig. 5w, x).

Post-hoc analysis revealed a significant increase in Aβ-positive cells in the STZ-Veh group compared to control groups. This increase was attenuated in the STZ-CBD group, with expression levels restored close to near-control values (*p* < 0.001) in both CA1 and DG regions. Similarly, in both the CA1 and DG regions, STZ-Veh rats exhibited a significantly higher number of p-Tau-positive cells compared to all other groups (*p* < 0.001).

A two-way ANOVA of the Aβ/p-Tau cell ratio in the CA1 and DG subregions revealed no significant effects of STZ [CA1: F(1,16) = 1.14, ns; DG: F(1,16) = 0.079, ns], CBD [CA1: F(1,16) = 3.14, ns; DG: F(1,16) = 1.909, ns], or STZ×CBD interaction [CA1: F(1,16) = 0.174, ns; DG:F(1,16) = 0.004, ns], suggesting that the Aβ/p-Tau ratio remained stable across both ACSF and STZ conditions, regardless of CBD administration (Fig. 5y, z).

## Discussion

We demonstrate that CBD prevents cognitive and social impairments, as well as hippocampal AD-related markers (Aβ accumulation, p-tau, TREM2, and ApoEɛ4) induced by STZ. It also reduces STZ-induced increases in hippocampal pro-inflammatory markers (TNF-α, NFkB1, IL-1β) and alters CB1/CB2 mRNA levels. Blockade of CB1r with AM251, but not CB2r with AM630, prevents CBD’s beneficial effects in object location and social tasks in STZ-treated rats. This suggests that CBD’s protective effects in the STZ AD model are likely due to its impact on CB1r activity.

As an initial step, this study examined the involvement of neuroinflammatory and endocannabinoid markers in the STZ model and was therefore conducted in males. Yet, we plan to investigate CBD treatment in females in future studies. The fact that the STZ model is typically applied only to males is a bias that limits the model’s representativeness [34].

### The effects of CBD on the behavioral phenotypes in STZ rats

Chronic CBD treatment prevents STZ-induced cognitive and social deficits, consistent with prior research showing similar outcomes in various models [35]. CBD has protective effects in neurodegenerative diseases, potentially reducing AD-related pathology, alleviating AD symptoms, and slowing cognitive decline [34, 35]. CBD also prevents memory deficits in Aβ-injected mice [19, 36], improves learning and reduces Aβ accumulation in female APPxPS1 mice [37, 38], and enhances recognition memory in pharmacological AD rat models [39].

No differences in anxiety-like behavior or motor function were observed. The rat model used here represents sporadic AD, which mimics late-onset AD. Anxiety and depression are more pronounced in early-onset AD [40]. Conflicting results on motor function have been reported in AD rodent models with some studies showing no locomotion deficits in the ICV-STZ model [41, 42].

### The effects of CBD on AD-related markers in STZ rats

Chronic CBD treatment reduced STZ-induced increases in Aβ protein levels, p-Tau, TREM2, and ApoEɛ4 mRNA expression, suggesting preventive effects on AD-related markers. While Aβ and tau pathology are key neuropathological hallmarks of AD [43], and their elevation has been documented in the STZ model [43–45], research on CBD’s impact on these markers is limited, mainly to in vitro models [46] [47, 48]. Our study provides the first evidence of CBD’s protective effects on Aβ and Tau in the STZ model.

TREM2 and ApoEɛ4 are markers of disease-associated microglia while some AD models report increased TREM2 expression [49–51], others show a decrease [52, 53]. These discrepancies likely reflect TREM’s stage-dependent role in AD, with low levels reducing proinflammatory cytokines in early stages and exacerbating neuroinflammation in later stages [54], as observed in our study.

Hippocampal expression of AD-related markers was significantly correlated with cognitive and social impairments, as previously suggested in AD and MCI patients [55, 56] and animal models [57–59]. Increased Aβ and p-tau levels in the CA1 and DG regions were associated with both cognitive and social deficits. Additionally, elevated TREM2 and ApoEε4 expression showed region-specific associations—correlating with cognitive deficits in the CA1 and social deficits in the DG. Evidence from rodent studies supports a role for TREM2 in modulating social behavior, likely through its effects on microglial function and synaptic development [60–62].

In our study, CBD modulated ApoEɛ4 expression in the DG, with no significant effect in the CA1. The ApoE gene, particularly the ε4 allele, is well-established as a major risk factor for AD, influencing amyloid and tau pathology, cognitive function, neurodegeneration, and immune responses [63, 64]. However, research on the interaction between ApoE and CBD is limited, with no studies directly examining CBD’s impact on ApoE levels in AD models.

Importantly, our immunohistochemical findings demonstrate that STZ-induced pathology is associated with increased Aβ and p-Tau accumulation in both the CA1 and DG and that CBD treatment reduced the expression of both markers, suggesting a potential neuroprotective effect.

### The effects of CBD on neuroinflammation in STZ rats

CBD prevented the STZ-induced increase in pro-inflammatory markers TNF-α and NFkB1 in the CA1 and DG and IL-1β in the CA1, highlighting the role of neuroinflammation in its therapeutic effects. TNF-α is typically low in healthy adults but significantly elevated in AD, where it accelerates cognitive decline [65–67]. NF-kB1 regulates pro-inflammatory gene expression and is implicated in inflammatory and neurodegenerative diseases [68, 69]. IL-1β, a central pro-inflammatory marker in AD, also influences disease risk [66, 70].

Changes in hippocampal neuroinflammatory markers were correlated with cognitive impairments (CA1: TNF-α, NF-κB1, IL-1β; DG: TNF-α, NF-κB1) and social deficits (CA1: TNF-α,IL-1β; DG: TNF-α, NF-κB1). Negative social interactions increase pro-inflammatory cytokines such as TNF-α [71], while positive social support reduces them [72]. Interestingly, while CA1 NF-κB1 mRNA expression was not significantly associated with social behavior, a significant effect was observed in the DG, suggesting a region-specific role of NF-κB1 in modulating social deficits in the STZ model. The DG is increasingly recognized for its role not only in cognitive functions but also in affective and social behavior, including stress regulation, novelty processing, and social memory [73, 74]. NF-κB signaling in the hippocampus has been linked to inflammatory responses that impair synaptic plasticity and behavior, and its upregulation in the DG may directly affect circuits underlying social behavior [75].

Clinical studies have linked IL-1β gene variants to cognitive performance in the elderly [76], and elevated IL-1β levels are associated with impaired social behavior in rats after proinflammatory stress [77]. The observed negative correlation between IL-1β levels in the CA1 region and both cognitive performance and social behavior aligns with previous studies demonstrating that elevated CA1 IL-1β contributes to hippocampal neuroinflammation, impairs memory function [78], and promotes inflammation-induced social withdrawal [79].

IL-1β mRNA expression in the DG was not significantly altered across experimental conditions, reflecting region-specific regulation of IL-1β signaling within the hippocampus. Recent studies have highlighted that, while the DG expresses neuronal IL-1 receptors, its response to IL-1β differs from other hippocampal subregions [80, 81]. Our study found no effect of STZ on IL-6 levels, consistent with findings that IL-6 is less frequently elevated in AD [82].

### The effects of CBD on CB1 and CB2 genes in STZ rats

In the CA1, CBD prevented the STZ-induced CB1 downregulation and CB2 upregulation, aligning with studies showing decreased CB1 and increased CB2 expression in postmortem AD brain tissues [83]. CB1r and CB2r play distinct roles in AD, with CB1 deficiency linked to cognitive impairment and exacerbated symptoms in mouse AD models [84–86]. CB1r protects against Aβ-induced toxicity, reduces gliosis, and improves spatial memory [87].

Conversely, research and post-mortem studies of Alzheimer’s patients show increased CB2r expression associated with Aβ and neuroinflammation [88, 89]. CB2 genetic deletion has been linked to reduced microglia-mediated neuroinflammation and improved spatial memory in mice [90, 91].

Interestingly, STZ upregulated CB1r mRNA expression in the DG. This opposing pattern may reflect a region-specific compensatory response, as CB1r upregulation in the DG is thought to reduce hyperexcitability and promote resilience under neural activity or damage [92, 93]. Additionally, inherent differences in endocannabinoid signaling and receptor distribution across hippocampal subregions may further contribute to these distinct expression profiles.

Changes in CA1 cannabinoid receptor expression correlated with impaired cognitive function (CB1 and CB2) and social deficits (CB2). These findings align with studies linking reduced CB1 expression to cognitive dysfunction in Parkinson’s models and AD postmortem brain tissues [94, 95] and highlighting CB2’s role in cognitive [96] and social [97, 98] functions.

### The effects of CB1 and CB2 receptor blockade following chronic CBD administration on the behavioral phenotypes in STZ rats

AM251, but not AM630, blocked CBD’s preventive effects on OL and social interaction tasks in STZ rats, though neither affected CBD’s impact on NOR. AM251 alone enhanced performance in STZ rats, consistent with previous studies showing improved NOR performance without affecting social behavior or anxiety [99–101]. Its therapeutic effects on memory may relate to the high density of PFC-CB1r [102, 103]. In AD models, AM251 has been shown to block the protective memory effects of ACEA [104], impair spatial memory, and increase tau mRNA expression in the Aβ toxin model of AD [105]. Conversely, it enhances spatial memory in ischemia‐induced cognitive impairment models [106]. These varied effects likely depend on dosage, model, timing, co-administration, brain region, and age [103, 107]. In contrast, AM630 consistently impairs cognitive and social functions [98, 106, 108].

The perirhinal cortex (PRC) plays a pivotal role in object recognition and is implicated in AD [109]. The STZ model is known to disrupt PRC function, which in turn impairs performance on learning-related tasks [110]. Also, in aged rats, the expression of CB1r is increased in the PRC and related brain areas [111]. Hence, the involvement of different brain structures encoding location (e.g., the hippocampus) and recognition (e.g., the PFC and the PRC) memories may account for the differential effects of AM251 on these types of memory [9].

### Low dose CBD administration

The doses of CBD (0.1 or 1 mg/kg) used in this study were relatively low compared to those commonly reported in the literature. Previous studies have employed a wide range of CBD doses from 0.05–0.25 mg/kg [26], 1 mg/kg [112] up to 50 mg/kg [29] in behavioral and biochemical experiments. CBD exhibits a biphasic effect, where low and high doses can lead to different, and sometimes opposing, physiological and behavioral outcomes [113, 114]. Studies indicate that lower doses of CBD tend to exert anti-inflammatory effects, whereas higher doses are often required for pronounced analgesic effects [115]. Additionally, dose-dependent interactions with different receptor systems, including CB1, CB2, TRPV1, and 5-HT1A, may contribute to variations in efficacy [116]. Future studies should further explore the therapeutic window of CBD to optimize its potential benefits and minimize variability in experimental outcomes.

## Conclusions

Our findings suggest that CBD protects against STZ-induced cognitive and social deficits, hippocampal neuroinflammation, and AD-related pathology, with CB1r playing a key role in its therapeutic effects. As current AD treatments are limited, our study highlights CBD as a promising candidate, demonstrating for the first time that a low dose can prevent behavioral and molecular deficits in a rodent model of sporadic AD. By targeting neuroinflammation and endocannabinoid pathways, CBD may help prevent cognitive decline and neuropathological changes in AD.

## Supplementary information

Supplemental material

## Data Availability

The authors declare that the data supporting the findings of this study are available within the paper and its Supplementary Information files. Should any raw data files be needed in another format they are available from the corresponding author upon reasonable request.
