# Supplementary material for: Cannabidiol prevents cognitive and social deficits in a male rat model of Alzheimer’s disease through CB1 activation and inflammation modulation
Source: Neuropsychopharmacology. 2025 Aug 26;50(13):1916–27. doi: 10.1038/s41386-025-02213-0 (PMC12603115; doi:10.1038/s41386-025-02213-0)
Supplement: Supplementary file 1 — Supplemental material [file 41386_2025_2213_MOESM1_ESM.docx]

Supporting Material for

**Cannabidiol Prevents Cognitive and Social Deficits in a Male Rat Model of Alzheimer’s Disease through CB1 Activation and Inflammation Modulation**

Roni Shira Toledano ^1,2^ and Irit Akirav^1,2, *^

^1^ School of Psychological Sciences, Department of Psychology, University of Haifa, Haifa 3498838, Israel

^2^ The Integrated Brain and Behavior Research Center (IBBRC), University of Haifa, Haifa, 3498838 Israel

*[iakirav@psy.haifa.ac.il](mailto:iakirav@psy.haifa.ac.il) (I.A.)

**Supplementary Methods**

**Detailed materials and methods**

***Animals***

Adult male Sprague-Dawley rats (two months old) were obtained from Envigo Laboratories (Jerusalem, Israel) and group-housed at 22±2°C under a 12-hour light/dark cycle (lights on at 07:00), with ad libitum access to food and water. The study was approved by the University of Haifa Ethics and Animal Care Committee (approval number: UoH-IL-2207-164-4) and adhered to NIH guidelines for minimizing pain and discomfort.

***Behavioral Tests***

Rats underwent all tests in a fixed order, spaced 24 hours apart, under dim lighting (15–20 lx) between 0900 and 1600 hours.

**Open Field Test (OFT)**

The OFT assesses locomotion and anxiety-like behavior. Rats explored a 50 × 50 cm black arena for 15 minutes. Locomotion (distance traveled) and freezing were recorded. The time spent in the arena’s center during the first 5 minutes measured novelty-related anxiety. The arena was cleaned with 70% ethanol between trials, and data were analyzed using EthoVision XT14 software (Noldus Information Technology, Wageningen, Netherlands).

**Object Location (OL) & Novel Object Recognition (NOR) Tasks**

The OL and NOR tasks assess spatial and visual recognition and working memory. After 2 days of habituation to the OFT arena, rats underwent a Sample Phase exploring two identical objects for 5 minutes. In the Test Phase, after a 5-minute inter-trial interval (ITI), one object was moved to a new location (OL) or replaced with a novel object (NOR). Exploration time was recorded for 5 minutes and a discrimination index (DI=TN/(TN+TF) was calculated (TN = novel place/object exploration time, TF = familiar place/object exploration time). Object locations were counterbalanced, and objects and the arena were cleaned with 70% ethanol between trials. Different copies of objects were used during the sample and test phases.

**Social Interaction Test (SIT)**

The SIT measures social interest. After 5 minutes of habituation in the OF arena, a same sex “partner” rat was introduced into the test arena, without prior placement in a holding cage. The interactions were recorded for 5 minutes. Social (e.g., sniffing, physical touch, getting under/on top) and non-social (e.g., self-grooming, remaining alone) behaviors were analyzed by a blinded experimenter, and a sociability index (social behavior time/total time) was calculated.

***Biochemical and Molecular Analyses***

**Western Blotting (WB)**

Based on [31]: Protein levels were measured using the bicinchoninic acid (BCA) Protein Assay Kit (Thermo Fisher Scientific, Waltham, MA, USA) following the manufacturer's protocol. Antibodies used included Anti-tau pS396 (79 kDa; 1:500; Abcam, Cambridge, UK; ab109390 [EPR2731]); Anti-tau (50 kDa; 1:10000; Abcam ab76128 [EP2456Y]); and anti Aβ (35 kDa; 1:200; Abcam ab201060 [Moc64]). β‐actin (45 kDa; 1:1000; Cell Signaling Technology, Danvers, MA, USA; #5125 [13E5]) served as a loading control.

**Quantitative Real-Time PCR (qRT-PCR)**

Based on [32,33]: mRNA (1000 ng) was converted to cDNA using the qScript cDNA Synthesis Kit (Quanta Biosciences, Gaithersburg, USA). Real-Time SYBR Green qRT-PCR was performed with specific primers (Quanta Biosciences) on a StepOne PCR system (Applied Biosystems). Fold change was calculated via the ddCt method, using HPRT as the reference gene. Primers for TNF-α, NF-κB1, IL-1β, IL-6, CB1r, CB2r, ApoEɛ4, and TREM2were synthesized by Agentek (Tel Aviv, Israel) and validated by standard curve and melting curve analysis. mRNA primer sequences are provided in Table S1.

***Immunohistochemistry procedure***

**Tissue fixation**

Animals were anesthetized by injection of ketamine/domitor (75/0.5 mg/kg, subcutaneously) and transcardially perfused with ∼250 ml of cold 0.01 M PBS solution, followed by 250 ml of 4% paraformaldehyde in 0.01M PBS. Brains were excised and postfixed in a fixative solution containing 30% sucrose and 4% paraformaldehyde in 0.01 M PBS for 48 h at 4°C. Following post-fixation, brains were frozen at −80°C until sectioning.

40 μm brain sections were sliced with a cooled cryostat (Leica, CM1900). Slices were washed with 0.01 M PBS and then blocked for 1h at room temperature with 0.01 M PBS containing 3% BSA. Sections were then incubated overnight at 4°C with Anti-tau pS396 (1:500;ab109390) and anti-Aβ (1:1000; ab201060) primary antibodies (Abcam, UK).

After 3 washes in 0.01 M PBS, Alexa488 (Molecular Probes) secondary antibodies diluted 1:1000 in 0.01 M PBS were applied consecutively for 1.5h each at room temperature. After two PBS 0.01 M washing steps, sections were mounted onto Super Frost-coated slides with Slow Fade antifade medium (Invitrogen). Slides were kept in the dark at 4°C. Slides were scanned using a Nikon A1R confocal microscope. A slide scanner SLIDEVIEW VS200 (Olympus/Evident) was used for imaging with a 20x objective. OlyVia software was used for cell counting and image extraction. In addition, the images of brain sections, whole depth Z-stacks, were taken on a Nikon confocal microscope with a Yokogawa CSU-W1 spinning disk. An objective of 40x with a short working distance was used.

**Supplementary Results**

**Statistics for the Open Field Test (OFT)**

**For Exp.1/Figure.1: Effects of Chronic CBD Administration on the Behavior of Rats with ICV-STZ Induced AD**

In the OFT, for distance traveled (Figure 1d), freezing levels (Figure 1e), and time spent in the center (Figure 1f), the two-way ANOVA [STZ×CBD (2×2)] revealed no significant effects of STZ [distance: F(1,40)=1.446, ns; freezing: F(1,40)=1.232, ns; time in center: F (1,40)=00.0, ns], CBD [distance: F(1,40)=3.237, ns; freezing: F(1,40)=10.848, ns; time in center: F(1,40)=0.814, ns], or the STZ×CBD interaction [distance: F(1,40)=0.828, ns; freezing: F(1,40)=0.077, ns; time in center: F(1,40)=0.018, ns]. The findings suggest that STZ did not cause any changes in motor function or anxiety-like behavior.

**For Exp.2/Figure.4: Effects of CB1 and CB2 Receptor Blockade on Behavior Following Chronic CBD Administration in Rats with ICV-STZ-Induced AD**

In the OFT, no significant group effects were observed for distance traveled (Figure 4d), freezing levels (Figure 4e), or time spent in the center (Figure 4f), as determined by one-way ANOVA [distance traveled: F(6,32)=1.494, ns; freezing: F(6,32)=2.088, ns; time spent in the center: F (6,37)=1.294, ns].

**Figure S4:**

***Comparison of the Behavioral Effects of Chronic Administration of Two CBD Doses in Rats with ICV-STZ-Induced AD***

In a preliminary study, we examined the effect of two low doses of CBD (0.1. mg/kg and 1 mg/kg) on performance in emotional and cognitive tests in STZ-treated animals.

In the OL task (Figure S4a), a one-way ANOVA revealed significant group effects [F(2,20)=33.811, p<0.001]. Post-hoc comparisons indicated that the DI was significantly higher in both the STZ+CBD 0.1 mg and STZ+CBD 1 mg groups compared to the STZ+Veh group (p<0.001), suggesting that both doses of CBD similarly affected performance in the OL task in STZ rats.

In the NOR task (Figure S4b), a one-way ANOVA revealed significant group effects [F(2,20)=40.599, p<0.001]. Post-hoc comparisons indicated that the DI was significantly higher in both the STZ+CBD 0.1 mg and STZ+CBD 1 mg groups compared to the STZ+Veh group (p<0.001), suggesting that both doses of CBD similarly affected performance in the NOR task in STZ rats.

In the SIT (Figure S4c), a one-way ANOVA revealed significant group effects [F(2,20)=179.168, p<0.001]. Post-hoc comparisons indicated that the sociability index was significantly higher in both the STZ+CBD 0.1 mg and STZ+CBD 1 mg groups compared to the STZ+Veh group (p<0.001), suggesting that both doses of CBD similarly affected performance in the SIT in STZ rats.

In the OFT, no significant group effects were observed for distance travelled (Figure S4d), freezing levels (Figure S4e), or time spent in the center (Figure S4f), as determined by one-way ANOVA [distance traveled: F(2,20)=1.42, ns; freezing: F(2,20)=0.216, ns; time spent in the center: F (2,20)=1.074, ns].

**Supplementary Figures**

**Figure S1: Diagram of the Experimental Design**


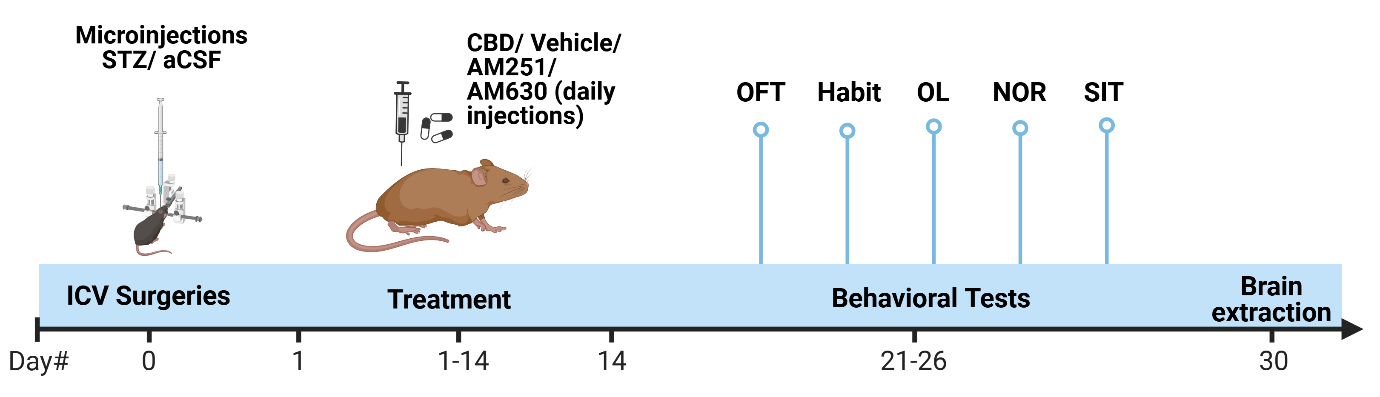
Adult male rats received an intracerebroventricular (ICV) injection of either STZ (10 μL, 3 mg/kg) or aCSF on Day 0. From Days 1 to 14, they were treated daily with either Vehicle, CBD (0.1 mg/kg), the CB1 receptor antagonist AM251 (0.3 mg/kg), or the CB2 receptor antagonist AM630 (1 mg/kg) for 14 consecutive days. Behavioral assessments were conducted between Days 21 and 26 in the following sequence: motor and emotional functions (Open Field Test, OFT), cognitive performance (Object Location, OL, and Novel Object Recognition, NOR, tests), and social behavior (Social Interaction Test, SIT). On Day 30, brain samples were collected for protein analysis of Aβ and Tau phosphorylation and mRNA expression of TNF-α, NF-κB1, IL-1β, IL-6, CB1r, CB2r, ApoEɛ4 and TREM2 in the hippocampal CA1 and dentate gyrus (DG) regions.

**Figure S2:** **Effects of Chronic CBD Administration on Total Exploration Time in the Object Location and Novel Recognition Tasks (Supplementary to Exp.1/Figure1)**

(a) A two-way ANOVA showed no significant effects in the exploration time of STZ [F(1,40)=4.008, ns], CBD [F(1,40)=1.567, ns], or the STZ×CBD interaction [F(1,40)=1.364, ns] during the OL sample phase.

(b) A significant effect in exploration time of STZ [F(1,40)=11.861, p<0.01], with no significant effects of CBD [F(1,40)=2.654, ns] or the STZ×CBD interaction [F(1,40)=1.598, ns] during the OL test phase. Post-hoc comparisons indicated that the aCSF+CBD group had a significantly higher total exploration time during the test phase compared to the STZ+Veh group (p<0.01).

(c) No significant effects of STZ [F(1,40)=2.95, ns], CBD [F(1,40)=0.057, ns], or the STZ×CBD interaction [F(1,40)=0.067, ns] was found during the NOR sample phase.

(d) No significant effects in exploration time of STZ [F(1,40)=1.119, ns], CBD [F(1,40)=2.931, ns], or the STZ×CBD interaction [F(1,40)=0.032, ns] were found during the NOR test phase.

These findings suggest that the total exploration time during the sample and test phases did not differ between groups.

**Figure S3: Social and Non-social Behaviors in the SIT Task (Supplementary to Exp.1/Figure 1)**

1. Sniffing: A two-way ANOVA revealed a significant effect of STZ [F(1,40)=5.085, p<0.05] and STZ×CBD interaction [F(1,40)=9.083, p<0.01] with no significant effect of CBD [F(1,40)=0.23, ns]. Post-hoc comparisons indicated that the STZ-Veh group exhibited significantly reduced sniffing time during the test compared to the aCSF-Veh group (p<0.001) and the STZ-CBD group (p<0.05).
2. Physical Touch: A two-way ANOVA revealed a significant effect of STZ [F(1,40)=3.873, p<0.05], CBD [F(1,40)=23.971, p<0.001] and STZ×CBD interaction [F(1,40)=5.55, p<0.05]. Post-hoc comparisons indicated that the STZ-Veh group exhibited significantly reduced physical touch time during the test compared to the aCSF-Veh (p<0.01), aCSF-CBD (p<0. 01) and the STZ-CBD (p<0.001) groups.
3. Getting Under/On-top: A two-way ANOVA revealed no significant effect of STZ [F(1,40)=0.21, ns], with significant effect of CBD [F(1,40)=27.736, p<0.001] and STZ×CBD interaction [F(1,40)=22.464, p<0.001]. Post-hoc comparisons indicated that the STZ-Veh group exhibited significantly reduced getting under/on-top time during the test compared to all other groups (p<0.001).
4. Non-Social: A two-way ANOVA revealed a significant effect of STZ [F(1,40)=6.074, p<0.05], CBD [F(1,40)=32.055, p<0.001] and STZ×CBD interaction [F(1,40)=21.779, p<0.001]. Post-hoc comparisons indicated that the STZ-Veh group exhibited significantly increased non-social behavior time during the test compared to all other groups (p<0.001).

**Figure S4:** **Comparison of the Behavioral Effects of Chronic Administration of Two CBD Doses in Rats with ICV-STZ-Induced AD**


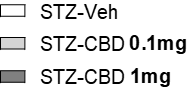

Animals in the STZ-Veh group exhibited the following impairments compared to the STZ+CBD 0.1 mg and STZ+CBD 1 mg groups: (a) deficits in the object location task, (b) reduced performance in the novel object recognition task, and (c) decreased sociability in the social interaction task. In contrast, (d) distance traveled, (e) freezing levels, and (f) time spent in the center during the open field test showed no significant differences between the groups. (n=5-10 per group), ***p<0.001.

**Figure S5:** **Representative Western blot images (Supplementary to Exp.1/Figure 2)**

**CA1**

aCSF-CBD

STZ-Veh

aCSF-CBD

aCSF-Veh

STZ-CBD


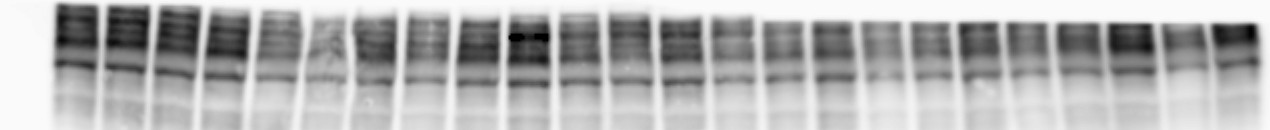


**Tau**


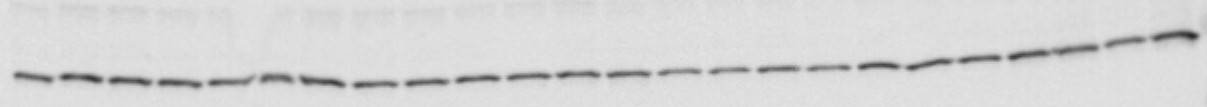


**Amyloid- β**


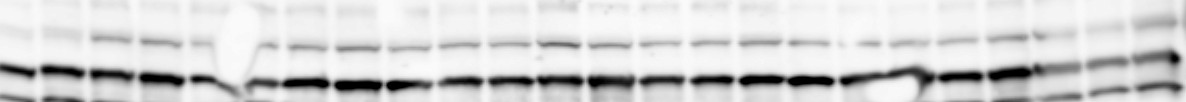


**β–Actin**

**DG**


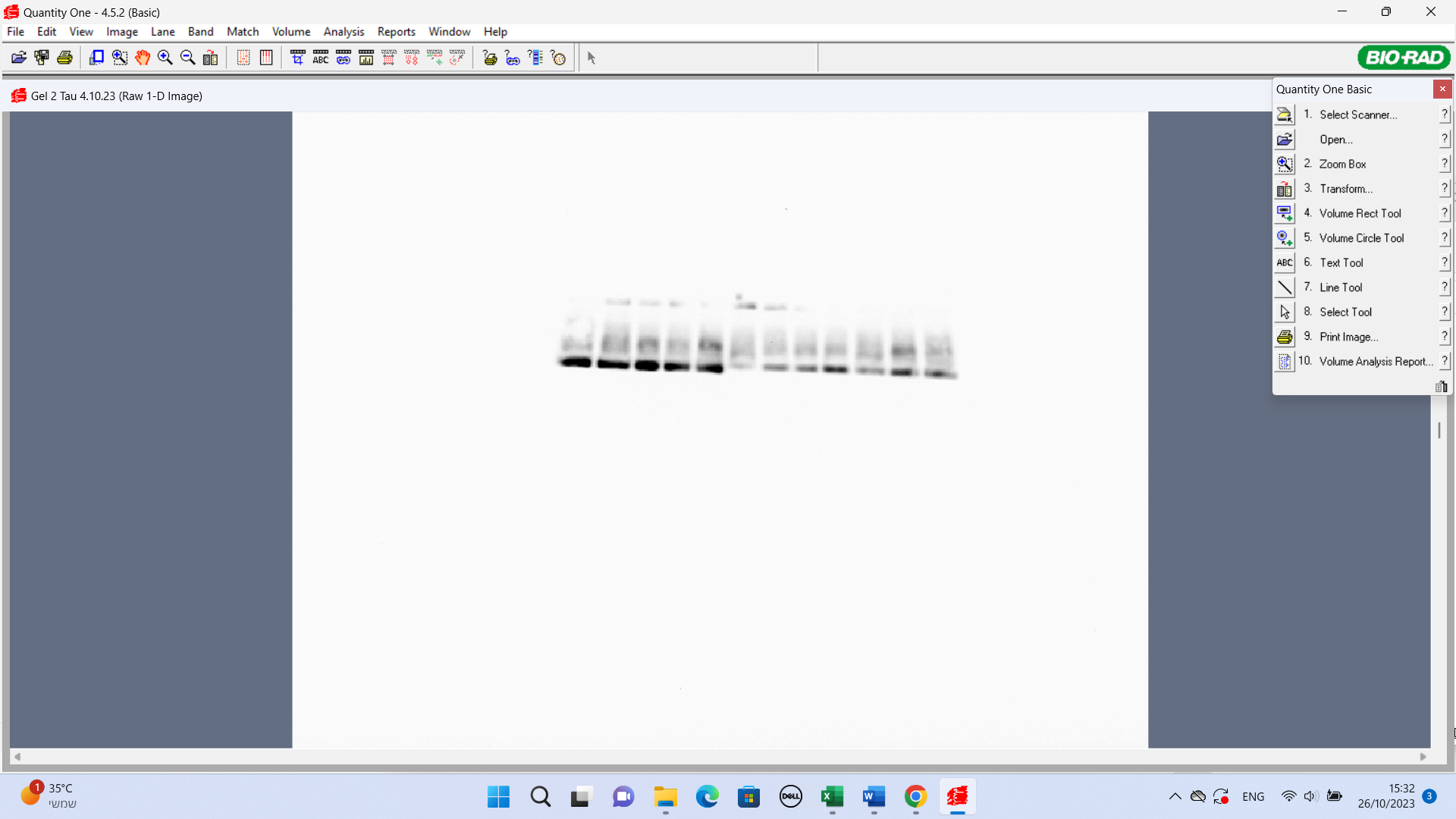

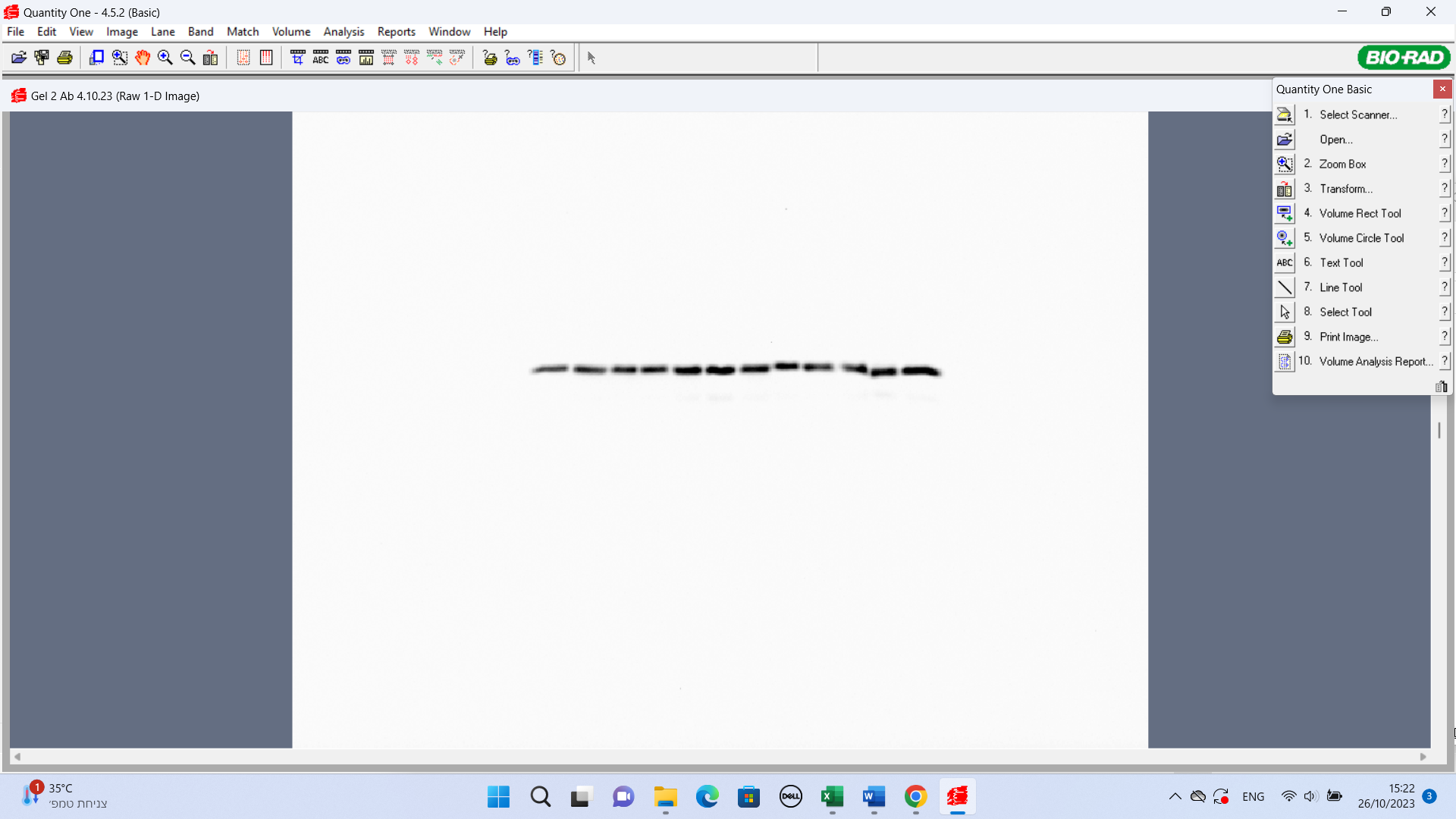

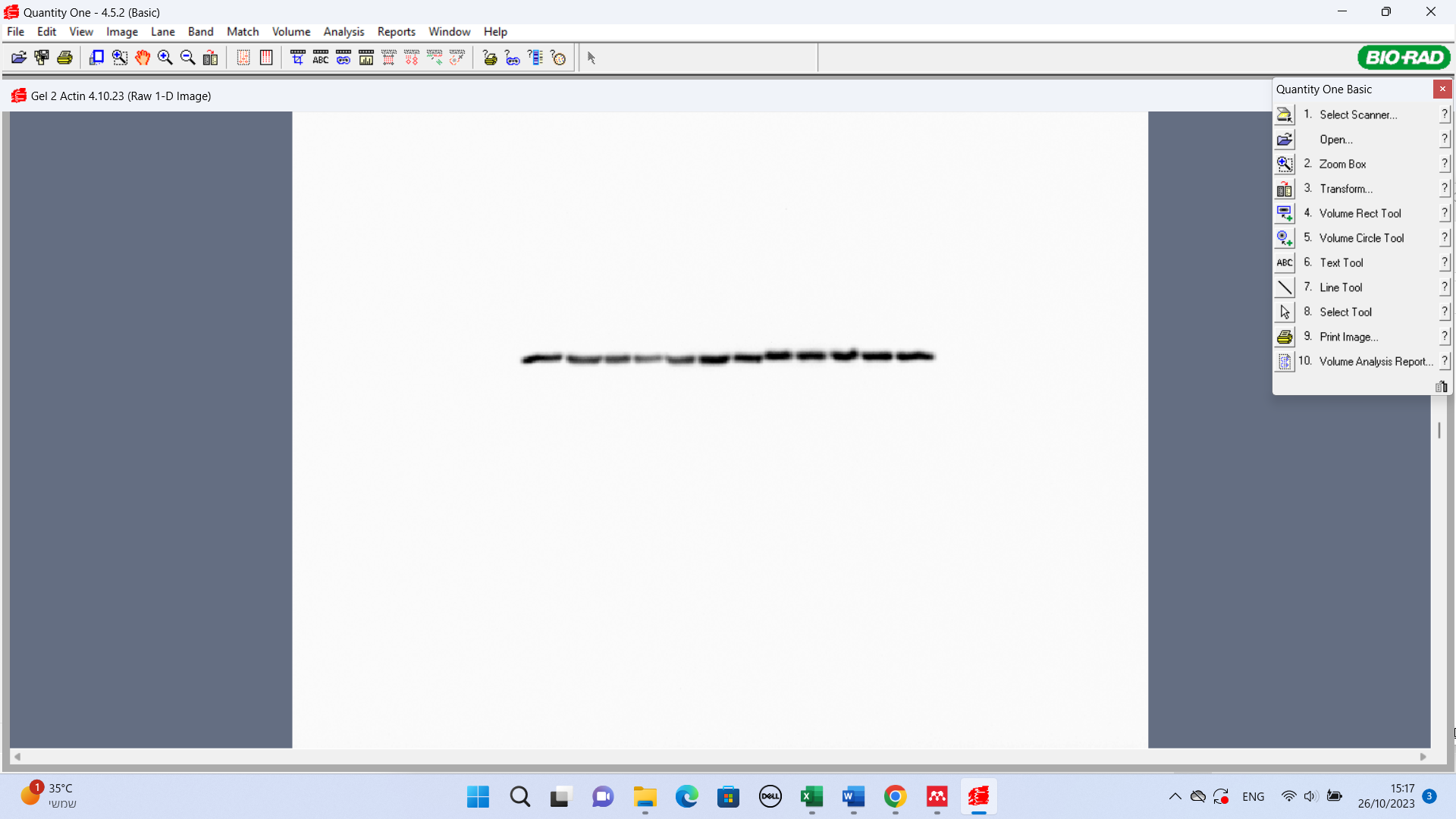


STZ-CBD

STZ-Veh

aCSF-CBD

aCSF-Veh

**Tau**

**Amyloid- β**

**β–Actin**

**Figure S6:** **Effects of Chronic CBD Blockers on Total Exploration Time in the Object Location and Novel Recognition Tasks (Supplementary to Exp.2/Figure4)**


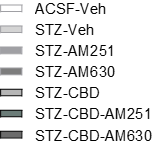

A one-way ANOVA revealed no significant group differences in total exploration time during the (a) OL task sample (F(6,42)=0.742, ns), (b) OL task test (F(6,42)=1.941, ns), (c) NOR task sample (F(6,42)=0.695, ns), or (d) NOR task test (F(6,42)=1.381, ns), indicating comparable performance across all groups in both tasks.


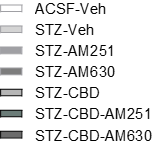
**Figure S7: Social and Non-social Behaviors in the SIT Task (Supplementary to Exp.2/Figure 4)**

1. Sniffing: A one-way ANOVA revealed significant group differences in sniffing time (F(6,41)=36.143, p<0.001). Compared to the STZ+CBD, STZ+CBD+AM630 and aCSF+Veh groups, the STZ+CBD+AM251 group showed reduced sniffing time in the SIT. In contrast, the STZ+CBD+AM630 group exhibited improved performance compared to the STZ+Veh, STZ+AM251, STZ+AM630, and STZ+CBD+AM251 groups.
2. Physical Touch: A one-way ANOVA revealed significant group differences in physical touch time (F(6,41)=33.988, p<0.001). Compared to the STZ+CBD, STZ+CBD+AM630 and aCSF+Veh groups, the STZ+CBD+AM251 group showed reduced physical touch time in the SIT. In contrast, the STZ+CBD+AM630 group exhibited improved performance compared to the STZ+Veh, STZ+AM251, STZ+AM630, and STZ+CBD+AM251 groups.
3. Getting Under/On-top: A one-way ANOVA revealed significant group differences in getting under/on-top behavior time (F(6,41)=2.923, p<0.05). Compared to the STZ+CBD+AM630 and aCSF+Veh groups, the STZ+CBD+AM251 group showed reduced getting under/on-top behavior time in the SIT. In contrast, the STZ+CBD+AM630 group exhibited improved performance compared to the STZ+Veh, STZ+AM251, STZ+AM630, and STZ+CBD+AM251 groups.
4. Non-Social: A one-way ANOVA revealed significant group differences in non-social behavior time (F(6,41)=38.219, p<0.001). Compared to the STZ+CBD, STZ+CBD+AM630 and aCSF+Veh groups, the STZ+CBD+AM251 group showed higher non-social behavior time in the SIT. In contrast, the STZ+CBD+AM630 group exhibited improved performance compared to the STZ+Veh, STZ+AM251, STZ+AM630, and STZ+CBD+AM251 groups.

**Supplementary Tables**

**Table S1**: rtPCR mRNA primer sequences

| mRNA | Protein Name | Forward primer | Reverse primer |
| --- | --- | --- | --- |
| hprt1 | HPRT | 5′CGCCAGCTTCCTCCTCAG3′ | 5′ATAACCTGGTTCATCATCACTAATCAC3′ |
| tnf | TNF-α | 5’AAATGGGCTCCCTCTCATCAGTTC3’ | 5’CTGCTTGGTGGTTTGCTACGAC3’ |
| nfkb1 | NF-κB1 | 5’GAGCTGCCCCATCTTCAAC3’ | 5'ATTTGCCCAGTTCCGAAAGGATC3' |
| Il1β | IL-1β | 5′GCTGTGGCAGCTACCTATGTCTT3′ | 5′GTCACAGAGGACGGGCTCTTC3′ |
| Il6 | IL-6 | 5′CTTCCAAACTGGATATAACCAGG3’ | 5′CTTCACAAACTCCAGGTAGAAAC3′ |
| cnr1 | CB1 | 5′CACCCATGGCTGAGGGTTC3′ | 5′CTGCAAGGCCATCTAGGATCGA3′ |
| cnr2 | CB2 | 5′GCCTGCAACTTCGTCATCTTC3′ | 5′TGCCGATCTTCAACAGGAA3′ |
| Apoe | APOE4 | 5′TGGGTGCAGACGCTTTCT3′ | 5′TCCTCCATCAGTACCGTCAGTTC3′ |
| Trem2 | TREM2 | 5′GTGCTGGAGGACCCTCTA3′ | 5′GGTGCTGTGCTCCACTTG3′ |

**Correlations**

**Table S2: Pearson Correlation Coefficients** **Between Hippocampal AD-Related Markers and Behavior**

Pearson bivariate correlation tests were conducted to examine the association between cognitive function, social behavior, and AD-like markers (amyloid β, tau, ApoEε4, and TREM2) in the DG and CA1.

In the OL task, negative correlations were observed with amyloid β (r=-0.545, p<0.01) and tau (r=-0.689, p<0.001) in the DG, as well as with ApoEɛ4 (r=-0.443, p<0.05) and TREM2 (r=0.-625, p<0.01) in the CA1. In the NOR task, negative correlations were found with amyloid β (r=-0.541, p<0.01) and tau (r=-0.664, p<0.01) and with ApoEɛ4 (r=-0.489, p<0.05) and TREM2 (r=-0.524, p<0.05) in the DG, and with ApoEɛ4 (r=-0.470, p<0.05) and TREM2 (r=-0.570, p<0.01) in the CA1. For sociability in the SIT, negative correlations were observed with amyloid β (r=-0.532, p<0.01) and tau (r=-0.637, p<0.01) in the DG, and with ApoEɛ4 (r=-0.443, p<0.05) and TREM2 (r=0.-625, p<0.01) in the CA1 (Table S2). These findings suggest that elevated levels of hippocampal AD-related markers are associated with impaired performance in the OL, NOR and SIT tasks.

**Table S2. Pearson correlation coefficients between hippocampal AD-like markers and behavioral measures**

|  | Object Location  Discrimination Index (DI) | Novel Object Recognition  Discrimination Index (DI) | Social Interaction  Sociability Index (SI) |  |
| --- | --- | --- | --- | --- |
| Amyloid β CA1 | **r= -.816**** | **r= -.597**** | **r= -.650**** |  |
|  | **p= 0.001** | **p= 0.003** | **p= 0.001** |  |
| Amyloid β DG | **r= -.545**** | **r= -.541**** | **r= -.532**** |  |
|  | **p= 0.003** | **p= 0.004** | **p= 0.004** |  |
| Tau CA1 | **r= -.866**** | **r= -.828**** | **r= -.714**** |  |
|  | **p= 0.001** | **p= 0.001** | **p= 0.001** |  |
| Tau DG | **r= -.689***** | **r= -.664**** | **r= -.637**** |  |
|  | **p= 0.001** | **p= 0.002** | **p= 0.003** |  |
| ApoEɛ4 CA1 | **r= .-443*** | **r= .-470*** | r= 0.171 |  |
|  | **p= 0.044** | **p= 0.036** | p= 0.458 |  |
| ApoEɛ4 DG | r= .-412 | r= .-270 | **r= -0.489*** |  |
|  | p= 0.071 | p= 0.250 | **p= 0.029** |  |
| TREM2 CA1 | | **r= .-625**** | **r= .-570**** | r= 0.416 |
|  |  | **p= 0.002** | **p= 0.007** | p= 0.061 |
| TREM2 DG | | r= .-418 | r= .-325 | **r= .-524*** |
|  |  | p= 0.075 | p= 0.175 | **p= 0.021** |

**Table S3: Pearson correlation coefficients between hippocampal neuroinflammation markers and behavioral measures**

Pearson bivariate correlation tests were conducted to examine the associations between cognitive function, social behavior, and the mRNA expression of neuroinflammatory markers in the CA1 and in the DG (TNF-α, NF-kB1, IL-1β and IL-6).

In the OL task, negative correlations were observed with TNF-α in the CA1 (r=-0.671, p<0.001) and in the DG (r=-0.581, p<0.05). Similarly, negative correlations were observed with NF-kB1 in the CA1 (r=-0.483, p<0.05) and in the DG (r=-0.437, p<0.05). Negative correlations were also observed with IL-1β in the CA1 1β (r=-0.695, p<0.001). In the NOR task, negative correlations were found with TNF-α in the CA1 (r=-0.624, p<0.001) and in the DG (r=-0.439, p<0.05). Additionally, negative correlations were found with NF-kB1 in the CA1 (r=-0.554, p<0.01) and in the DG (r=-0.524, p<0.05). Negative correlations were also found with IL-1β in the CA1 (r=-0.675, p<0.001). For sociability in the SIT, a negative correlation was observed with TNF-α in the CA1 (r=-0.505, p<0.01) and in the DG (r=-0.598, p<0.05). A negative correlation was observed with NF-kB1 in the DG (r=-0.568, p<0.05). A negative correlation was also observed with IL-1β in the CA1 (r=-0.494, p<0.05) (Table S3).

These findings suggest that elevated levels of TNF-α, NF-κB1, and IL-1β in the CA1 region are associated with impaired performance in both the OL and NOR tasks. In the DG region, OL and NOR deficits were linked to increased TNF-α and NF-κB1 expression. Furthermore, social interaction deficits were correlated with elevated TNF-α and IL-1β levels in CA1, and with increased TNF-α, NF-κB1, and IL-1β expression in the DG.

**Table S3. Pearson correlation coefficients between neuroinflammation markers and behavioral measures**

|  | Object Location  Discrimination Index (DI) | Novel Object Recognition  Discrimination Index (DI) | Social Interaction  Sociability Index (SI) |  |
| --- | --- | --- | --- | --- |
| TNF-α CA1 | **r=-.671***** | **r= -.624***** | **r= -.505**** |  |
|  | **p= 0.001** | **p= 0.001** | **p= 0.007** |  |
| TNF-α DG | | **r=-.581*** | **r= -.439*** | **r= -.598*** |
|  |  | **p= 0.018** | **p= 0.048** | **p= 0.014** |
| NF-κB1 CA1 | **r= -.483*** | **r= -.554**** | r= -0.279 |  |
|  | **p= 0.017** | **p= 0.006** | p= 0.186 |  |
| NF-κB1 DG | | **r= -.437*** | **r= -.524*** | **r= -.568*** |
|  |  | **p= 0.014** | **p= 0.045** | **p= 0.027** |
| IL-1β CA1 | **r= -.695***** | **r= -.675***** | **r= -.494*** |  |
|  | **p= 0.001** | **p= 0.001** | **p= 0.017** |  |
| IL-1β DG | | r= -.322 | r= -.246 | r= -.435 |
|  |  | p= 0.224 | p= 0.358 | p= 0.092 |
| IL-6 CA1 | r= -0.031 | r= -0.276 | r= -0.229 |  |
|  | p= 0.893 | p= 0.239 | p= 0.319 |  |
| IL-6 DG | | r= -0.407 | r= -0.248 | r= -0.191 |
|  |  | p= 0.147 | p= 0.393 | p= 0.512 |

**Table S4: Correlations between CB1 and CB2 gene expression and behavior**

Pearson bivariate correlation tests were conducted to examine the association between cognitive function, social behavior, and the mRNA expression of CB1 and CB2 cannabinoid receptors in the CA1 and in the DG regions.

In both the OL and NOR tasks, a positive correlation was found with CB1 receptor (OL: r=0.447, p<0.05; NOR: r=0.573, p<0.01) and a negative correlation with CB2 receptor (OL: r=-0.663, p<0.001; NOR: r=-0.677, p<0.001). For sociability in the SIT, a negative correlation was observed with CB2 (r=-0.632, p<0.001) (Table S4). These findings suggest that decreased levels of cnr1 and increased levels of cnr2 were associated with impaired performance in the OL and NOR tasks. Increased levels of cnr2 were also associated with deficits in social interaction.

**Table S4. Pearson correlation coefficients between CB1 and CB2 gene expression and behavioral measures**

|  | Object Location  Discrimination Index (DI) | Novel Object Recognition  Discrimination Index (DI) | Social Interaction  Sociability Index (SI) |  |
| --- | --- | --- | --- | --- |
| CB1 (cnr1) CA1 | **r=.447*** | **r=.573**** | r= 0.140 |  |
|  | **p= 0.029** | **p= 0.004** | p= 0.515 |  |
| CB1 (cnr1) DG | | r=-.467 | r=-.468 | r= -0.012 |
|  |  | p= 0.058 | p= 0.078 | p= 0.967 |
| CB2 (cnr2) CA1 | **r= -663***** | **r= -.677***** | **r= -.632***** |  |
|  | **p= 0.001** | **p= 0.001** | **p= 0.001** |  |
| CB2 (cnr2) DG | | r= -.280 | r= -.266 | r= -.441 |
|  |  | p= 0.246 | p= 0.271 | p= 0.059 |
